# Intraoperative Pontine Infarction: A Hidden Challenge

**DOI:** 10.1155/2012/807398

**Published:** 2012-04-03

**Authors:** Nicholas Marcanthony, Ehab Farag

**Affiliations:** Anesthesiology Institute, Cleveland Clinic, Cleveland, OH 44195, USA

## Abstract

Apneusis, or apneustic respirations, is characterized by an abnormal breathing pattern involving gasping and the inability to fully expire. A loss of gag reflex and other cranial nerve deficits are also often accompanied with these respiratory changes. In neurological intensive care units (NICUs), these respiratory and airway changes are not uncommon and have been well documented (Lee et al. 1976). These clinical changes are often associated with pontine trauma as it is the core pneumotaxic center in the brain stem. We describe the airway management of a patient with an acute, occult pontine infarct status post craniectomy and cervical laminectomy for decompression of known Chiari malformation in the postanesthesia care unit (PACU).

## 1. Introduction

The world Pons is derived from the Latin word for bridge. This is an appropriate name as its anatomical location allows it to functionally serve as a conduit for all the nuclei from the cerebrum and medulla. Specifically, it is the pneumotaxic center for the body. It regulates conversion of inspiration to expiration, including coordination of the vocal cords for proper air flow. Damage to this pneumotaxic center can result in apneustic respirations [1]. Recognition of this is critical because immediate airway management may be required.

## 2. Case Summary

 The patient was a 40-year-old woman with Chiari malformation Type I. Her other past medical history included hypertension, dyslipidemia, hypothyroidism, esophageal reflux, and active smoking. She underwent (presented to the PACU status post) craniectomy and cervical laminectomy for decompression.

 She was induced with midazolam 2 mg, fentanyl 1.5 mcg/kg, lidocaine 1 mg/kg, propofol 2 mg/kg, and rocuronium 0.5 mg/kg. Remifentanil infusion was run at 0.1 mcg/kg/min from intubation to emergence. Over the course of the 4-hour- and 50-minute case, an additional 50 mg of rocuronium and 100 mcg of fentanyl were used. Vancomycin and gentamicin were used for antibiotic prophylaxis. She was left intubated postoperatively for unresolved neuromuscular blockade. This prolonged blockage may have been related to the above combination of nondepolarizer and aminoglycoside. This pharmacological combination has been shown to prolong paralysis for several hours [2].

 The patient remained intubated and sedated in the recovery area for greater than two hours. A nerve stimulator was used to test for a train of four and confirmed a strong 4/4. The patient was given a full reversal consisting of neostigmine 50 mcg/kg and glycopyrrolate 10 mcg/kg. Sedation was turned off and the patient began to awake shortly thereafter.

 The patient was evaluated after she was completely awake. She was able to follow all commands, move all extremities, and had sustained head lift greater than 5 seconds. Her tidal volume was greater than 5 cc/kg, and her maximum inspiratory volume was greater than 12 cc/kg without tachypnea or difficulty.

 Almost immediately after extubation the patient began to exhibit signs of respiratory distress. Her work of breathing rapidly increased over the first few minutes. Stridorous respirations were audible after about five minutes. Not long thereafter the patient was visibly gasping for air.

 Ventilation was supported via bag-mask ventilation. Awake fiber-optic bronchoscopy was performed due possible difficulty and diagnostic advantages at time of intubation. Further evaluation, included consultation with ENT, CT and MRI studies, and admission to the neurological intensive care unit. MRI of the brain later revealed ischemic changes in the right basis pontis, dorsal right medulla, and cerebellar tonsils.

## 3. Discussion

The pneumotaxic center is responsible for coordinating the movement of the vocal cords synchronously with respirations. In a patient with an insidious infarct to the pons, such as our patient, this coordination does not exist [3].

 While the patient was intubated, her endotracheal tube was continuously maintaining patency, keeping the vocal cords open. With this aid, she had no clinically observable difficulty with her breathing. However, upon extubation, this aid was taken from the patient, and her respiratory distress ensued. There was a mismatch and discoordination between her ventilation and vocal cord movement leading to the described stridor and increased work of breathing.

 As an anesthesiologist, one of our key functions is airway management. Pontine infarction poses a particular difficultly in that one of its key sequela is masked whilst the patient is intubated. Because of this, after extubation, close observation and diligent airway management must be maintained. A low threshold for reintubation is necessary for patient safety and prevention of hypoxia.
